# Some of the Results of the Early Extraction of the Sixth Year Molar

**Published:** 1892-07

**Authors:** W. B. Conner

**Affiliations:** Akron, O.


### Some of the Results of the Early Extraction of the Sixth Year Molar.

BY W. B. CONNER, D. D. S., AKRON, O.

Read before the Northern Ohio Dental Society, Cleveland, May 1892.

Mr. President and Members of the Northern Ohio Dental Association: In the presentation of this paper, it will be my aim and purpose to show some of the changes that take place following the extraction of the sixth year molar during the developmental age of the maxilla and teeth.

I have no doubt there are some here who regard this subject threadbare, it may be however, but only in spots, for there are interesting facts to every question, if only pointed out.

During the development of the bones of the face and maxilla we are many times called upon to perform the disagreeable task of removing this enfeebled member, and our consciences often rebel at the thought, knowing what results will follow. Nevertheless with all our entreaties and kind solicitations, we are often obliged to extract the same, charging impatience and obstinacy to the patient or parent. Again where we have the confidence and willingness on the part of the patient, and retention is possible, some dentists are so wavering or vacillating as to what theory is best to pursue, they will reluctantly set aside their own judgment and be carried away by this or that author's vaunted or radical idea, and in the course of time bewail their folly.

The maxillary bones are unlike other bones in the body, in many respects, but are subject to the same laws of nature governing repair.

An excision or fracture of a shaft bone will not unite without producing a shortening. The same rule applies to all bones and flesh wounds. Also that destruction is always greatest in the direction of the least resistance.

It is popularly supposed that after this tooth has been extracted, that the second molar moves forward, and occupies its place. This is not altogether true, and here is where a mistaken theory has been misleading. In every case of early extraction of the first permanent molar the change that takes place in the jaws is contrary to the popular theory advocated.

There is no force brought to bear upon a second molar that would cause it to move forward. The second molar is held in its normal position by its antagonist, and does not move forward, instead, the change is wholly anterior to the same, and comprises a retrograde movement of the jaw.

The roots of the sixth year molar penetrate the true maxilla to about one half their entire length; where extraction is resorted to, the lesion involves not only the process, but far into the jaw proper.

We have exactly the same condition as we would find in a shaft bone where fracture has taken place, only in a less degree.

What would be the result if a fracture of a shaft bone were not supported by splints during the knitting together of the parts? Would not muscular contraction distort that joint, and produce an unsightly and impaired member?

If the rule applies to one should it not also to the other?

The part of the jaw implicated in extraction is consequently weakened, and at an early age when the bones are yielding, is much more susceptible to the pressure of muscles than at a more mature age.

The strongest and most unyielding part of the jaws, both upper and lower are posterior to the second bicuspids, but owing to the extent of bone involved in the extraction of this molar it is made the weakest, and the muscular pressure brought to bear upon the anterior portion of the jaw, forces the superior jaw backward and upward, and the lower jaw backward and downward.

The model of the upper jaw before you, illustrates better than I can the result of the force mentioned.

The bicuspids, canines and incisors, have not separated in their movement backward, but you will observe that after the bicuspids lost their antagonism they were forced inward by muscular contraction.

I do not wish to convey the idea that such changes take place after the fifteenth year, but have made these observations where extraction has occured prior to that time.

If the first molar is extracted before the second molar has erupted, we find the space closing up sooner, and to all appearances there has not been any retrograde movement on the part of the anterior teeth. But upon examination of the bite and jaws, we find a change, and a change not entirely at the expense of the jaw posterior to the lesion.

It has been taught in our colleges and journals, that we must expect the second molar to occupy the space left vacant, and our natural conclusion has been in harmony with these teachings.

It is contrary to all scientific reasoning to expect such a result, where extraction takes place at an age when the jaws are so yielding to the forces brought to bear upon them.

How often do we find a first molar moving forward to occupy the space left vacant after extracting the second bicuspid?

I have never observed one such instance. Is it not just as lame an argument to advance as that of a second molar moving forward to occupy the first molars space?

The models I have passed around are casts of my own mouth, showing the arrangement of the teeth and changes taken place.

You will readily see the absence of the first molars in both upper and lower jaws. The upper were extracted at about the tenth or eleventh year and the lower were left in the jaw until exfoliated, which was completed at the fifteenth or sixteenth year.

So the upper were lost before and the lower several years after the eruption of the second molars, bringing about the change presented.

We have in this case a decided shortening of the bite which is shown by the position of the occluding teeth, instead of an interlocking of the canines and bicuspids, which was the normal occlusion prior to the extraction. The superior canines and incisors are striking directly upon the incisive surfaces of the eight anterior lower teeth, proving conclusively a determined shortening of the bite.

There is also a loss of the use of six bicuspids, the four superior and two inferior.

I might say here that in all cases the lower jaw is not interfered with to near the extent of the upper, after extraction, owing no doubt to the fact that the inferior maxillary bone is ossified earlier in life and is of a stronger mold and texture offering more resistance.

I maintain that the superior maxillary in this case has been shortened to the extent of the width of the missing tooth extracted, not saying anything about the constriction in the width of the jaw.

The second inferior molar having erupted earlier than the superior, which is true in the majority of cases, gives it more prestige, having a firmer union with the jaw controlling the movement of its antagonist.

The result, you observe, holding the superior second molar in nearly its normal position, thereby prohibiting an anterior movement but not a rotary movement.

The forces brought to bear at this early age upon the superior teeth anterior to the first molar, might be regarded as insignificant.

We observe in our every day practice that the protrusions of the superior or inferior canines, if let alone, will in the course of a short time be adjusted to the arch and antagonistic teeth. The only force brought to bear is the weight of and contractions of the obicularis oris muscle with the assistance of the muscles of expression and mastication.

If the bones and process is so yielding at the time of the eruption of the canines, why should not the jaw be more so during the eruption of the second molars two years previous?

There is no doubt that if such changes as above mentioned come about at the age of twelve that the jaws have not become fully developed, and adhering to the law governing repair, the destruction is always greatest in the direction of the least resistance.

We have a retrograde movement in every case of early extraction of the sixth year molar.

In the superior maxillary the molar eminence adds its marked features to the face, its consequent absorption, its dire effect upon the stronger expression.

In every case of early extraction there is an entire loss of this protuberance and the muscles and surfaces over it become shortened and flattened with constriction of the face and in its vicinity.

There is a depression at the alæ nasi and a sunken condition of the bones, which occasionally extènd to the floor of the orbit. In the face of all the abnormalties produced, we have our journals full of this or that theory relative to the time when this tooth should be extracted. One writer claims the eighth year, another the tenth, and another the eleventh or twelfth, and all for what purpose—to bring about a better arrangement and condition of the teeth and for the benefit of humanity in general.

I am safe in making the assertion that nature has not produced as many irregularities as the early extraction of the sixth year molar has occasioned.

Does extracting this tooth at an early age preclude all possibility of an overcrowded condition?

Can we foretell the size, development and exact arrangement of the teeth and jaws far into the future?

If such prophetic knowledge is possessed by a few, the whole profession should know it.

Nature's plan of eruption is a most harmonious one.

When the first molar is erupted it acts as an anchor or foundation to the arch, always in the proper place, well adapted for the purpose intended, and in the course of time the second molar erupts, reinforced, as it were, in accordance with the eternal fitness of things.

Now are we, as a scientific profession going to improve upon nature's plan by removing the foundation, and dictate our plan of constructing a human jaw?

We should have not only one object in view when extracting, giving room for incoming teeth and temporary relief. The best safeguard a dentist can have is to preserve, if possible, all of the sixth year molars until that condition has come about when the bones of the face have become perfectly developed and features set—the second molars, bicuspids and canines held in position by firm occlusion.

Then, and not until then, do I consider it prudent for the first permanent molar to be removed.

Everything is conjecture and visionary when we extract this tooth at an early age, with such delusions as some authors advocate.

Our observations, if closely applied, will not direct or advise such a procedure.

The intelligence of the age demands of us all that our knowledge can avail, and it does not show that we are advancing when we resort to such practice as we have been taught to follow in the past.

